# Survivin counteracts the therapeutic effect of microtubule de-stabilizers by stabilizing tubulin polymers

**DOI:** 10.1186/1476-4598-8-43

**Published:** 2009-07-03

**Authors:** Chun Hei Antonio Cheung, Huang-Hui Chen, Ching-Chuan Kuo, Chi-Yen Chang, Mohane S Coumar, Hsing-Pang Hsieh, Jang-Yang Chang

**Affiliations:** 1 National Institute of Cancer Research, National Health Research Institutes, Tainan 704, Taiwan R.O.C; 2 Division of Biotechnology and Pharmaceutical Research, National Health Research Institutes, Zhunan, Miaoli County 350, Taiwan R.O.C; 3 Division of Hematology and Oncology, Department of Internal Medicine, National Cheng Kung University Hospital, Tainan 704, Taiwan R.O.C

## Abstract

**Background:**

Survivin is a dual function protein. It inhibits the apoptosis of cells by inhibiting caspases, and also promotes cell growth by stabilizing microtubules during mitosis. Over-expression of survivin has been demonstrated to induce drug-resistance to various chemo-therapeutic agents such as cisplatin (DNA damaging agent) and paclitaxel (microtubule stabilizer) in cancers. However, survivin-induced resistance to microtubule de-stabilizers such as *Vinca *alkaloids and Combretastatin A-4 (CA-4)-related compounds were seldom demonstrated in the past. Furthermore, the question remains as to whether survivin plays a dominant role in processing cytokinesis or inhibiting caspases activity in cells treated with anti-mitotic compounds. The purpose of this study is to evaluate the effect of survivin on the resistance and susceptibility of human cancer cells to microtubule de-stabilizer-induced cell death.

**Results:**

BPR0L075 is a CA-4 analog that induces microtubule de-polymerization and subsequent caspase-dependent apoptosis. To study the relationship between the expression of survivin and the resistance to microtubule de-stabilizers, a KB-derived BPR0L075-resistant cancer cell line, KB-*L30*, was generated for this study. Here, we found that survivin was over-expressed in the KB-*L30 *cells. Down-regulation of survivin by siRNA induced hyper-sensitivity to BPR0L075 in KB cells and partially re-stored sensitivity to BPR0L075 in KB-*L30 *cells. Western blot analysis revealed that down-regulation of survivin induced microtubule de-stabilization in both KB and KB-*L30 *cells. However, the same treatment did not enhance the down-stream caspase-3/-7 activities in BPR0L075-treated KB cells. Translocation of a caspase-independent apoptosis-related molecule, apoptosis-inducing factor (AIF), from cytoplasm to the nucleus was observed in survivin-targeted KB cells under BPR0L075 treatment.

**Conclusion:**

In this study, survivin plays an important role in the stability of microtubules, but not with caspases inhibition. Over-expression of survivin counteracts the therapeutic effect of microtubule de-stabilizer BPR0L075 probably by stabilizing tubulin polymers, instead of the inhibition of caspase activity in cancer cells. Besides microtubule-related caspase-dependent cell death, caspase-independent mitotic cell death could be initiated in survivin/BPR0L075 combination treatments. We suggest that combining microtubule de-stabilizers with a survivin inhibitor may attribute to a better clinical outcome than the use of anti-mitotic monotherapy in clinical situations.

## Background

Microtubules are protein filaments of cytoskeleton composed of α-tubulin and β-tubulin molecules [1,2]. In cells, microtubule filaments rapidly alternate between phases of growth and shrinkage (dynamic instability) during cell cycle. Since microtubules play crucial roles in the regulation of the mitotic apparatus, disruption of microtubules can induce cell cycle arrest in M phase, the formation of abnormal mitotic spindles, and final triggering of signals for apoptosis. The discovery that the cytotoxic activity of various compounds is through interference with the mitotic spindle apparatus has attracted much attention within the past two decades, and microtubules have become an attractive pharmacologic target for anticancer drug discovery [3,4]. Anti-mitotic compounds such as vincristine, vinblastine (microtubule-destabilizing *Vinca *alkaloid) and paclitaxel (microtubule-stabilizing taxane) have been developed to target cancers clinically [5-7]. Although the taxanes and *Vinca *alkaloids are effective for the management of different malignancies, their potential is limited by the development of multidrug resistance (MDR) [8,9]. MDR is multi-factorial, with one pathway leading to resistance mediated by over-expression of transmembrane efflux pumps, namely, the *M*_r _170,000 P-glycoprotein (P-gp170/MDR) and multidrug resistance protein (MRP) [10]. Therefore, there has been great interest in identifying novel microtubule inhibitors that can overcome various modes of resistance and have improved pharmacology profiles.

BPR0L075 [6-methoxy-3-(3',4',5'-trimethoxy-benzoyl)-1H-indole] is a novel synthetic compound discovered through research to identify new microtubule inhibitors in our laboratory. It is a heterocyclic Combretastatin A-4 (CA-4) analog, which is derived from the South African tree *Combretum caffrum*, and inhibits tubulin polymerization by binding to tubulin at the colchicine-binding site [11,12]. Unlike traditional microtubule inhibitors such as vincristine and paclitaxel, BPR0L075 is effective in suppressing cell growth of both MDR-positive and -negative tumor cell lines [12]. BPR0L075 also induces microtubule de-stabilization-related downstream processes such as phosphorylation of Bcl-2 and activation of caspase-3 [12]. *In-vivo*, BPR0L075 shows potent activity against the growth of xenograft tumors of the gastric carcinoma MKN-45, human cervical carcinoma KB, and KB-derived P-gp170/MDR-overexpressing KB-VIN10 cells in nude mice [12]. Although BPR0L075 is a promising anticancer compound that can be applied to the management of various malignancies, particularly for patients with MDR-related drug resistance, its effectiveness in patients with MDR-unrelated drug resistance is still unknown.

Survivin is a member of the inhibitors of apoptosis (IAPs). Other than the expression of MDR, expression of survivin has been related to the causation of cancer-drug resistance in various studies [13-15]. At the molecular level, survivin is a bifunctional protein that acts as a suppressor of apoptosis and plays a central role in cell division. It has been suggested that survivin, possibly the mitochondrial fraction instead of the cytosol fraction, inhibits apoptosis through interference with caspases [16-18]. A study using surface plasmon resonance spectroscopy showed that survivin directly binds to caspase-3 and caspase-7 with nanomolar affinity [16]. In confirmation, myc-tagged survivin bound caspase-3 and caspase-7 in immunoprecipitation studies [16]. Survivin also promotes cell survival through interference with cell cycle-related kinases and microtubule networks. Survivin binds to centromeres on a para-polar axis during prophase/metaphase, relocates to the spindle midzone during anaphase/telophase, and disappears at the end of telophase [19]. Over-expression of survivin reduced centrosomal microtubule nucleation and suppressed both microtubule dynamics instability in mitotic spindles and bidirectional growth of microtubules in midbodies during cytokinesis [20]. In addition, it has been shown that intracellular loading of a polyclonal antibody to survivin induced microtubule defects and resulted in formation of multipolar mitotic spindles [21]. Although over-expression of survivin has been suggested to cause resistance to various chemotherapeutic compounds in cancers, survivin-induced resistance to microtubule de-stabilizers such as *Vinca *alkaloids and cochicine-related compounds in cancers was seldom demonstrated in the past [22]. Furthermore, the question remains as to whether survivin plays a dominant role in processing cytokinesis or inhibiting caspases activity in cells treated with anti-mitotic compounds. In fact, down-regulation of survivin did not induce massive cell death in HeLa cells [23]. However the process of cytokinesis was heavily impaired by the same treatment [23]. In this study, we have generated a microtubule de-stabilizer BPR0L075-resistant cell line and shown for the first time that down-regulation of survivin enhances sensitivity to the microtubule de-stabilizing compound BPR0L075. We further describe the role of survivin in interfering sensitivity to the microtubule de-stabilizer, BPR0L075, in human oral carcinoma cells.

## Results

### Over-expression of survivin in both BPR0L075/colchicine-treated cancer cells and BPR0L075-resistant cancer cells

**Figure 1:**
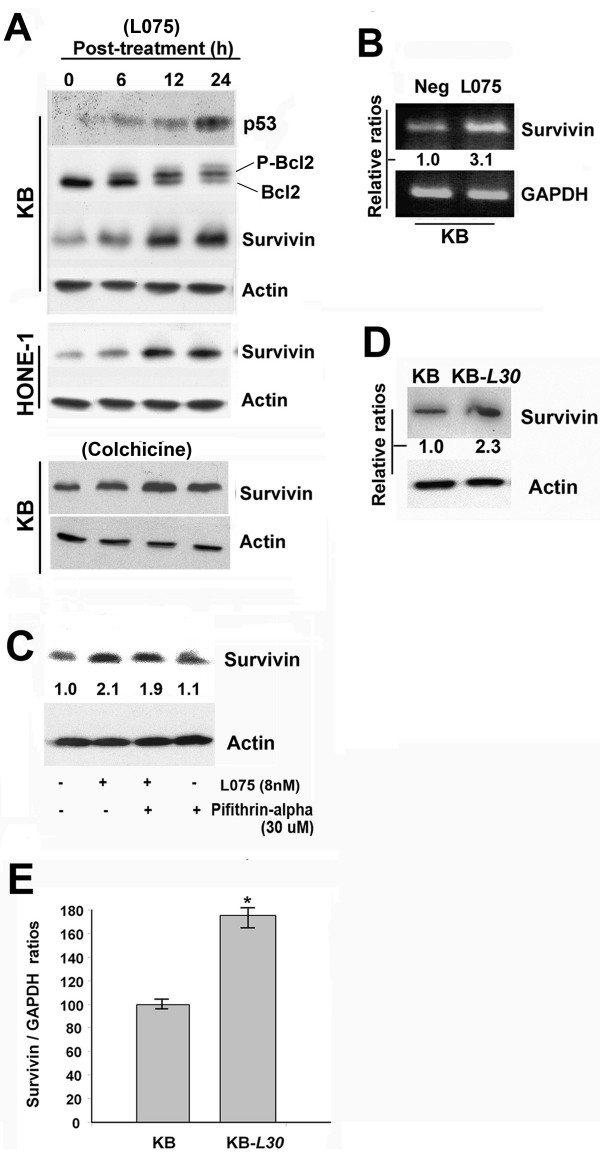
**BPR0L075 induces over-expression of survivin in human KB and HONE-1 cancer cells**. (**A**) Cells were treated with 8 nM of BPR0L075 and 10 nM of colchicine for 6 h, 12 h and 24 h. Cell lysate were extracted and proteins were resolved by SDS-PAGE. Western blot analysis was performed as described in Materials and Methods. (**B**) KB cells were treated with 8 nM of BPR0L075 for 24 h and total RNA was extracted. Level of survivin mRNA was determined by RT-PCR. GAPDH was included for the use of semi-quantization. Relative band intensity was shown. (**C**) KB cells were incubated with 8 nM of BPR0L075 for 24 h with/without pifithrin-α pre-incubation. Cell lysate were extracted and proteins were resolved by SDS-PAGE. Western blot analysis was performed as described in Materials and Methods. Relative band intensity was shown. (**D**) KB-*L30 *cells over-expresses survivin. Total cell lysate of KB and K-*L30 *cells was extracted and proteins were resolved by SDS-PAGE. Western blot analysis was performed as described in Materials and Methods. Relative band intensity was shown. (**E**) Total mRNA of KB and KB-*L30 *cells was extracted. Level of survivin mRNA was determined by real-time PCR. Relative survivin to GAPDH ratios were shown. A statistically significant difference in the level of survivin mRNA between KB and KB-*L30 *cells is denoted by "*", (p < 0.001).

Over-expression of survivin in response to anti-cancer treatments has been demonstrated previously [24]. Here, we examined the level of survivin expression in response to BPR0L075 treatment in various cancer cell lines. Human p53-wildtype KB and p53-mutated HONE-1 cancer cell lines were used in this study. Cells were treated with 8 nM (IC_50 _value) of BPR0L075 for 24 h. Cell lysate was extracted and the expressions of various intracellular proteins were analyzed by western blotting. A time-dependent phosphorylation of Bcl2 in KB cells was shown and this result was consistent with our previously published data (Figure 1A) [12]. Both un-treated cell lines expressed survivin endogenously (Figure 1A). In addition, the treatment of BPR0L075 (8 nM) induced over-expression of survivin in a time-dependent manner in both cancer cell lines (Figure 1A). Surprisingly, over-expression of a survivin negative-regulator, p53, in response to BPR0L075 was also observed in KB cells in a time-dependent manner (Figure 1A).

KB cells were chosen for further *in vitro *investigation, considering that the same cell line was chosen in our previously published report [12]. To determine whether over-expression of survivin was unique to BPR0L075 treatment, KB cells were treated with 10 nM (IC_50 _value) of colchicine (microtubule destabilizer) and intracellular proteins were analyzed by western blotting. Cells incubated with colchicine also induced over-expression of survivin in a time-dependent manner (Figure 1A, bottom panel). FACS analysis was performed to determine whether BPR0L075-induced over-expression of survivin was caused by cell cycle arrest at the G_2_/M phase. KB cells were treated with various concentrations of BPR0L075 for 24 h and nucleus was stained with propidium iodide. Although high concentrations (≥ 16 nM, 2 × IC_50_) of BPR0L075 induced G_2_/M arrest, FACS analysis clearly showed no difference in the percentage of G_2_/M population between untreated-cells and cells treated with 8 nM (IC_50 _value) of BPR0L075 (Additional file 1).

To determine whether BPR0L075-induced over-expression of survivin in KB cells was caused by changes in the rate of survivin gene transcription, RT-PCR was performed. As was observed for the protein, the level of survivin mRNA transcripts was increased by approximately 3-fold in KB cells after 24 h of BPR0L075 incubation (Figure 1B). It has been suggested that p53 down-regulates the expression survivin [25]. To determine whether p53 plays a role in the BPR0L075-induced over-expression of survivin in KB cells, a p53 inhibitor pifithrin-α was used. Interestingly, western blot analysis revealed that co-incubation pifithrin-α was unable to increase the level of survivin expression in BPR0L075-treated KB cells (Figure 1C). Taken together, these results suggest that p53-indpendent over-expression of survivin may affect cancer cells susceptibility to BPR0L075-induced cytotoxicity.

A KB-derived BPR0L075-resistant cancer cell line, KB-*L30*, was recently generated in our laboratory. This drug-resistant cell line was shown to withstand 30 nM of BPR0L075 under culturing condition (data not shown). In addition, this specific cell line showed resistance to colchicine *in vitro *(data not shown). Under BPR0L075-free conditions, western blot analysis revealed that the baseline expression of survivin was increased by approximate 2.3-fold in KB-*L30 *cells, as compared to the drug-sensitive KB cells (Figure 1D). Real-time PCR also revealed that the level of survivin mRNA transcripts was significantly increased by approximate 80% (*p < 0.01) in the KB-*L30*, as compared to its drug-sensitive parental cells (Figure 1E). FACS analysis was performed to determine whether over-expression of survivin in KB-*L30 *was caused by cell cycle arrest at the G_2_/M phase. KB and KB-*L30 *cells cultured under BPR0L075-free coditions were stained with propidium iodide. FACS analysis clearly showed no significant difference in the percentage of G_2_/M population between KB (16.3%) and KB-*L30 *(16.5%) cells (Additional file 1). Taken together, the detection of high levels of survivin in the BPR0L075-resistant KB-*L30 *cells suggests that intracellular survivin may affect cancer cells susceptibility to BPR0L075-induced cytotoxicity.

### Down-regulation of survivin increases sensitivity to BPR0L075 and colchicine in cancer cells

**Figure 2:**
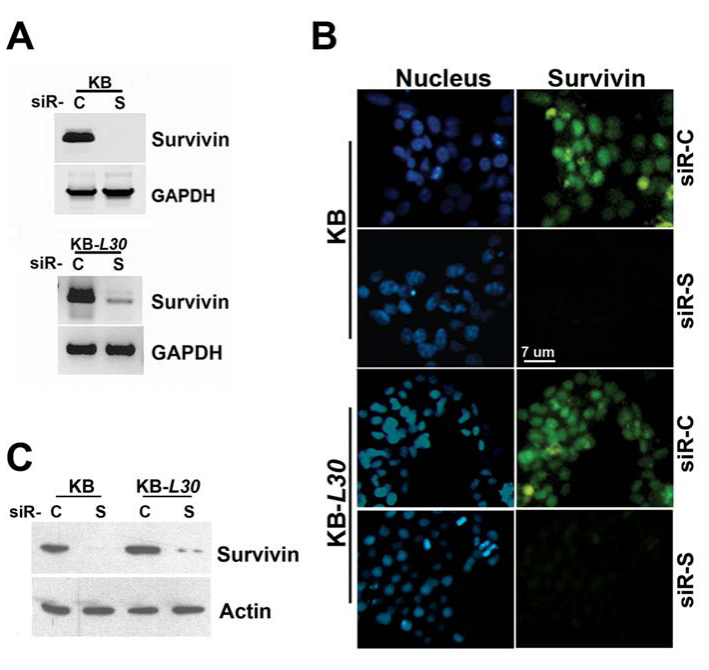
**Down-regulation of survivin in KB and KB-*L30 *cells by siRNA**. (**A**) Cells were seeded on 24-well plates overnight and transfected with siR-C (scramble control) or siR-S (survivin specific) for 36 h. Total RNA was extracted by Trizol reagent. RT-PCR was performed and PCR products were resolved by DNA electrophoresis. GAPDH was used as an internal control (**B**) Cells were seeded on 8-well chamber slides overnight and transfected with siR-C (scramble control) or siR-S (survivin specific) for 48 h. Cells were labeled with FITC-coupled anti-human survivin antibody and counter-stained with DAPI nucleus stain. Slides were analyzed using fluorescent microscopy. (**C**) Cells were seeded on 6-well plates overnight and transfected with siR-C or siR-S for 48 h. Cell lysate were extracted and proteins were resolved by SDS-PAGE. Western blot analysis was performed as described in Materials and Methods.

To investigate the sensitivity of cancer cells to the microtubule de-stabilizer, BPR0L075, after inhibition of survivin, siRNA was applied to down-regulate survivin in cancer cell lines, and the level of cell viability was examined. Cells were transfected with validated-siRNA oligomers, siR-C (scramble control) or siR-S (survivin specific) by liposomal reagent. Down-regulation of survivin mRNA transcripts by siRNA in both KB and KB-*L30 *cells after 36 h of transfection was confirmed by RT-PCR (Fig. 2A). Successful down-regulation of survivin protein by siR-S after 48 h of transfection was shown by immunofluorescence microscopy (Figure 2B). Western blot analysis further confirmed that survivin protein levels were successfully down-regulated after 48 h of siRNA transfection (Figure 2C).

**Figure 3:**
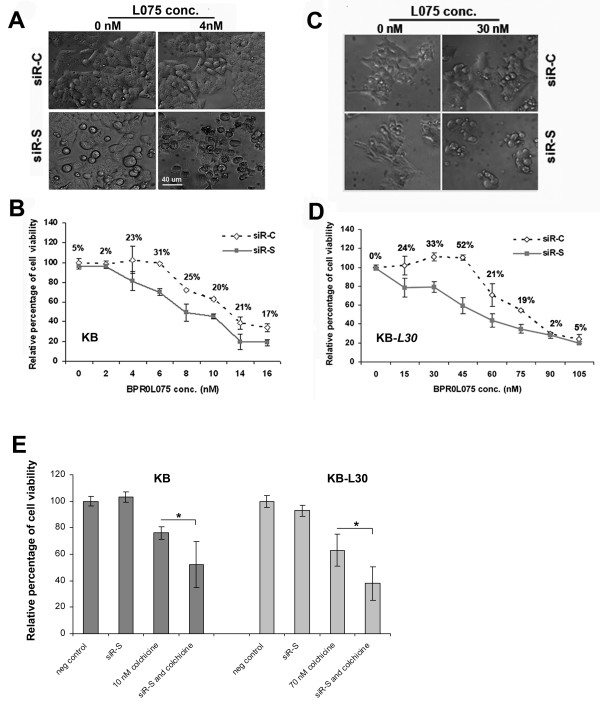
**Down-regulation of survivin increases sensitivity to BPR0L075 and colchicine in KB and KB-*L30 *cells**. KB and KB-*L30 *cells were seeded on 96-well plates overnight. Cells were pre-transfected with siR-C (scramble control) or siR-S (survivin specific) for 24 h and incubated with various concentrations of BPR0L075 for 48 h. Cells were analyzed by (**A and C**) phase-contrast microscopy and (**B and D**) MTT cell viability assay. Mean differences in cell viability between treatments at same dose of BPR0L075 were labeled on the graph. (**E**) KB and KB-L30 cells were transfected with siR-C (scramble control) or siR-S (survivin specific) for 24 h and incubated with 10 nM (IC_50 _value, KB) and 70 nM (IC_50 _value, KB-*L30*) of colchicine for 48 hours. Cells were analyzed by MTT cell viability assay. A statistically significant difference in the level of viability between cells treated with siR-S/BPR0L075 combination therapy and BPR0L075 monotherapy is denoted by "*", (p < 0.001).

Morphologically, the survivin-targeted KB cells became round and slightly enlarged in size (Figure 3A). Although down-regulation of survivin by siR-S induced morphological changes in KB cells under BPR0L075-free conditions (Figure 3A), the same treatment did not reduce cell viability (Figure 3B). Interestingly, down-regulation of survivin by siR-S enhanced the sensitivity to BPR0L075 in KB cells *in vitro*. Co-treatment of siR-S and BPR0L075 significantly reduced cell viability of KB by 17–31% among various concentrations of BPR0L075 *in vitro *as compared to the drug alone treatment (Figure 3B).

We further examined the possibility of restoring the sensitivity to BPR0L075 by down-regulation of survivin in KB-*L30 *cells. Phase contrast microscopy and cell viability studies showed that down-regulation of survivin did not induce KB-*L30 *cells cell death under BPR0L075-free conditions (Figure 3C and 3D). In contrast, down-regulation of survivin by siR-S induced massive cell death in KB-*L30 *cancer cells under normal culture conditions (30 nM of BPR0L075) (Figure 3C and 3D). Co-treatment of siR-S and BPR0L075 reduced the viability of KB-*L30 *cells by 20–52% among various concentrations of BPR0L075 *in vitro *as compared to the drug alone treatment (Figure 3D).

We have previously demonstrated that colchicine induced over-expression of survivin in KB cells (Figure 1A). To determine whether down-regulation of survivin by siRNA also increases the sensitivity to colchcine in KB and KB-*L30 *cells, cells co-treated with siR-C/siR-S and colchicine were analyzed by cell viability assay. Interestingly, down-regulation of survivin by siR-S enhanced the sensitivity to colchicine in both KB and KB-*L30 *cells *in vitro *(Figure 3E). Taken together, our results indicate that survivin plays an important role in the sensitivity to microtubule de-stabilizers, BPR0L075 and colchicine, in both KB and KB-*L30 *cells.

### Survivin interferes with microtubule dynamics

**Figure 4:**
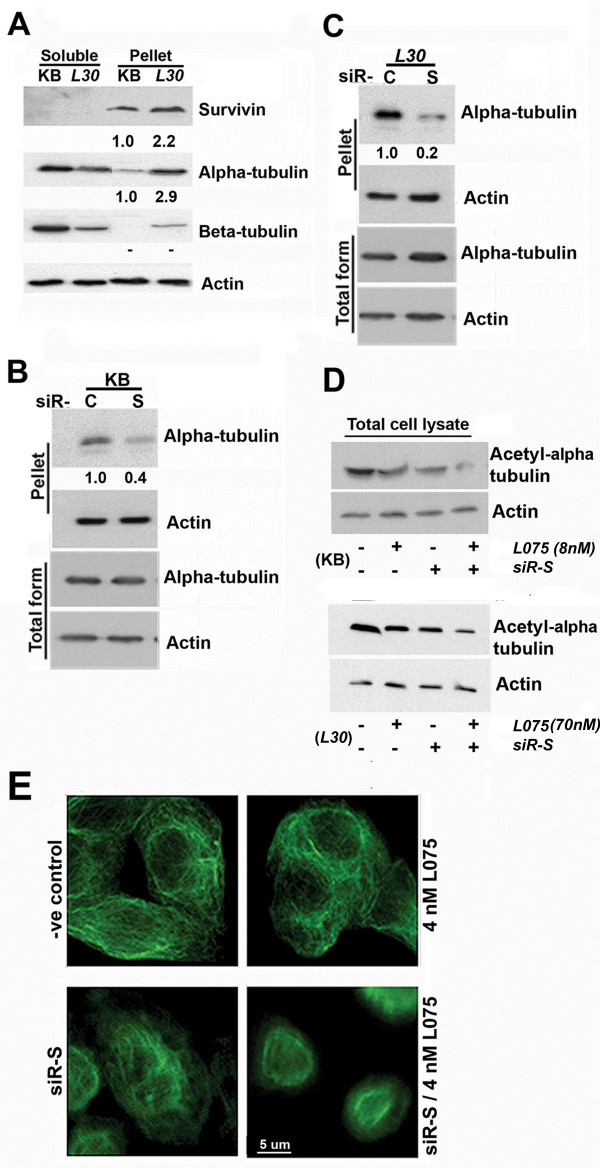
**Down-regulation of survivin changes the dynamics of microtubule networks**. (**A**) Supernatant and pellet fractions of un-treated KB and KB-*L30 *cells were extracted. Proteins were resolved by SDS-PAGE and analyzed by western blotting. Anti-actin was used as an internal control. Relative band intensities were shown. (**B and C**) KB and KB-*L30 *cells were transfected with siR-C (scramble control) and siR-S (survivin specific) for 48 h. Total lysate and pellet fractions of cells were extracted. Proteins were resolved by SDS-PAGE and analyzed by western blotting. Anti-actin was used as an internal control. Relative band intensity was shown. (**D**) Down-regulation of survivin reduced tubulin acetylation in KB and KB-*L30 *cells. Cells were transfected with siR-C or siR-S for 24 h and co-incubated with/without 8 nM (KB) and 70 nM (KB-*L30*) of BPR0L075 for 48 h. Total cell lysate of cells was extracted. Proteins were resolved by SDS-PAGE and analyzed by western blotting. Anti-actin was used as an internal control. (**E**) Down-regulation of survivin destabilizes the microtubule networks. KB cells were seeded on 8-well chamber slides. Cells were pre-transfected with siR-C (scramble control) or siR-S (survivin specific) for 24 h and co-incubated with 4 nM of BPR0L075 for 24 h. Cells were fixed and labeled with mouse anti-human α-tubulin antibody and FITC-coupled anti-mouse secondary antibody. Slides were analyzed by fluorescent microscopy.

BPR0L075-induced de-stabilization of microtubule has been shown in cancer cells [12]. On the other hand, stabilization of microtubule networks has been suggested to induce drug-resistance of tubulin de-stabilizating agents in HUVEC cells [26]. Therefore, microtubule polymerization status was investigated to determine whether survivin counteracts the effectiveness of BPR0L075 by interfering with the microtubule dynamics in KB cancer cells. Soluble and insoluble fractions of the cell lysate were extracted. Interestingly, survivin was not detected in the soluble fraction of both cell lines (Figure 4A). The amount of insoluble survivin in KB-*L30 *was increased by approximately 2.2-fold as compared to the BPR0L075-sensitive KB cells. In correlation, the amounts of insoluble α – and β-tubulin were also increased in KB-*L30 *cells (Figure 4A). In contrast, the amounts of soluble α – and β-tubulin were decreased in KB-*L30 *cells (Figure 4A). These results reveal a positive correlation between the expression of survivin and stabilization of microtubule.

Interaction between survivin expression and dynamic formation of the microtubule was shown by western blot analysis. Down-regulation of survivin by siR-S reduced the amount of insoluble α-tubulin in both KB and KB-*L30 *cells (Figure 4B and 4C). However, the same treatment did not induce any changes in the general expression of α-tubulin (Figure 4B and 4C). Tubulin acetylation is considered to be a marker of microtubule stabilization [27,28]. In our study, western blot analysis revealed that down-regulation of survivin by siR-S reduced the amount of intracellular acetylated α-tubulin in both KB and KB-*L30 *cells, as compared to siR-C treated control. The combination of siR-S and 8 nM of BPR0L075 further reduced the amount of acetylated α-tubulin, as compared to siR-S and drug alone treatment in KB cells (Figure 4D, top panel). In addition, combination of siR-S and 70 nM of BPR0L075 also reduced the amount of acetylated α-tubulin, as compared to siR-S and drug alone treatment in KB-*L3*0 cells (Figure 4D, bottom panel). Immunofluorescent microscopy was used to provide visualization of the microtubule networks in cells. Untreated KB cells showed intact microtubule networks (Figure 4E). In contrast, down-regulation of survivin by siR-S induced disorganization of the microtubule networks (Figure 4E). Furthermore, the combination of siR-S and 4 nM of BPR0L075 induced synergistic microtubule depolarization and changes in cell morphology (Figure 4E). These results indicate that survivin stabilizes the microtubule networks and possibly counteracts the therapeutic effect of microtubule de-stabilizer, BPR0L075, by stabilizing tubulin polymers.

### Inhibition of survivin enhances sensitivity to BPR0L075 via caspase-3/-7 dependent/independent mechanisms

BPR0L075-induced microtubule de-stabilization and subsequent activation of caspase-3 has been shown in KB cells previously [12]. Here, real-time caspase-3/-7 activity imaging was performed to determine whether de-stabilization of the microtubule networks by targeting survivin enhances the sensitivity to BPR0L075 in cancer cells via downstream caspase-3/-7 activations. Cells pre-transfected with siR-C and siR-S were incubated with BPR0L075 for various durations. The activities of caspases-3/-7 were measured with the MagicRed™ real-time caspase-3/-7 detection kit, and nuclei were counter-stained blue with a Hoechst dye.

**Figure 5:**
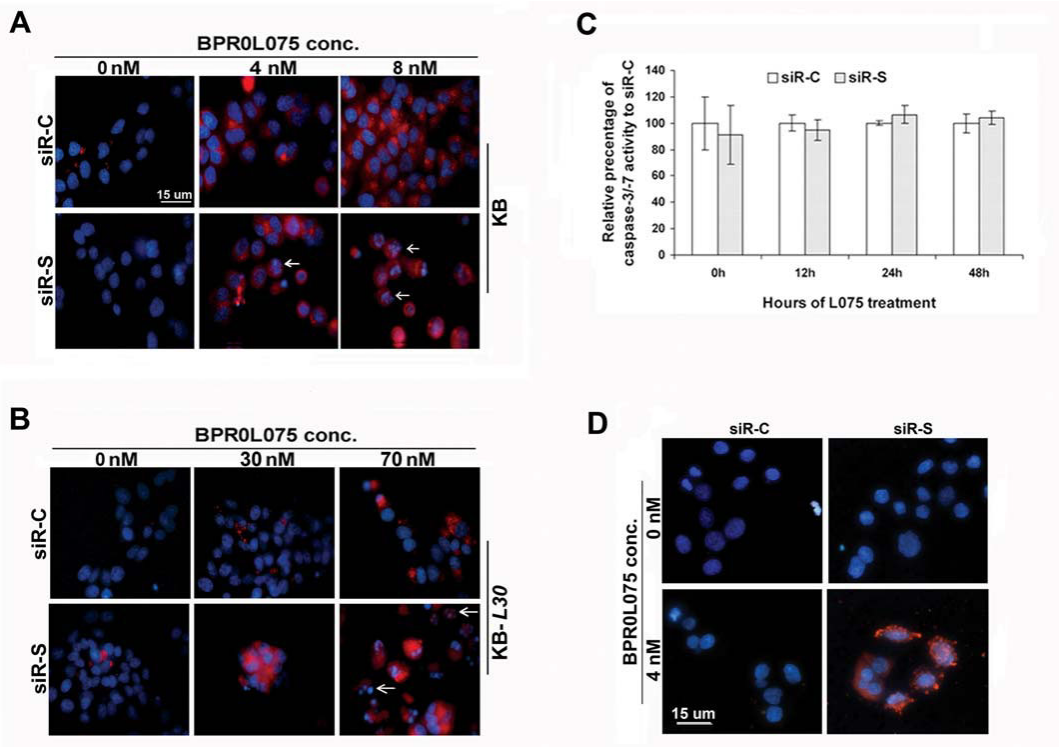
**Down-regulation of survivin with co-incubation of BPR0L075 enhances the activity of caspase-3 in KB-*L30*, but not KB cells**. (**A and B**) KB and KB-*L30 *cells were seeded on 8-well chamber slides overnight. Cells were pre-transfected with siR-C (scramble control) or siR-S (survivin specific) for 24 h and co-incubated with various concentrations of BPR0L075. MagicRed™-DEVD Real-time Caspase-3/-7 Activity kit (Immunochemistry Technologies LLC) was used. Activated-caspase-3/-7 was stained red and nucleus was counter-stained blue by Hoechst 33342. White arrows indicate cells with nucleus degradation. (**C**) Quantitative measurement of caspase-3/-7 activity. KB cells were seeded on 96-well plate and transfected with siR-C (scramble control) or siR-S (survivin specific) for 24 h. siRNA-treated cells were incubated with 4 nM of BPR0L075 for various times. Caspase-3/-7 activity was analyzed by the use of MagicRed™-DEVD real-time caspase-3/-7 activity kit with a 96-well plate-reader. (**D**) Analysis of DNA fragmentation by TUNEL assay. KB cells were pre-transfected with siR-C (scramble control) or siR-S (survivin specific) for 24 h and co-incubated with 4 nM of BPR0L075 for 48 h. DNA fragmentations were analyzed using the *In Situ *Cell Death Detection kit. Nucleus with DNA fragmentation was stained red.

In BPR0L075-free conditions, caspase-3/-7 activities were not detected in both cell lines (Figure 5A and 5B). KB-*L30 *cells were normally cultured in media with the presence of BPR0L075 (30 nM). In this condition, caspase-3/-7 activities were also not detected (Figure 5B, middle row). In contrast, high dose of BPR0L075 induced caspase-3/-7 activity in both KB (8 nM, IC_50 _value) and KB-*L30 *(70 nM, IC_50 _value) cells, as indicated by red fluorescence staining of the cytoplasm of cells (Figure 5A and 5B). Inhibition of survivin by siR-S did not induce caspase-3/-7 activities in both cell lines under drug-free conditions (Figure 5A and 5B). In addition, co-treatment of siR-S and 4 nM of BPR0L075 did not enhance caspase-3/-7 activities in KB cells, as compared to the BPR0L075 monotherapy at various time points (Figure 5C). Interestingly, down-regulation of survivin induced caspase-3/-7 activities in BPR0L075-cotreated KB-*L30 *cells (Figure 5B, middle row). Therefore, our results indicate that survivin directly or indirectly interferes with the caspase-3/-7 in KB-*L30*, but not in KB cells during BPR0L075 treatments.

Activation of caspase-3/-7 induces DNA fragmentation [29,30]. Nucleus degradation was observed with KB cells treated with 16 nM (twice of IC_50 _value) of BPR0L075 after 72 h (data not shown). Here, massive nucleus degradation was observed in the siR-S/BPR0L075-cotreated KB-*L30 *cells (Figure 5B, white arrows). In contrast, intact nucleus was shown in the drug alone treatment (Figure 5B). Although caspase-3/-7 activities were not increased in the combination treatment, increased degradation of nucleus was shown in KB cells, as compared to the drug alone treatment (Figure 5A, white arrows). The early appearance of DNA fragmentation in KB cells under combination treatment was further confirmed by TUNEL assay (Figure 5D).

### Down-regulation of survivin induces translocation of apoptosis-inducing factor in KB cells

**Figure 6:**
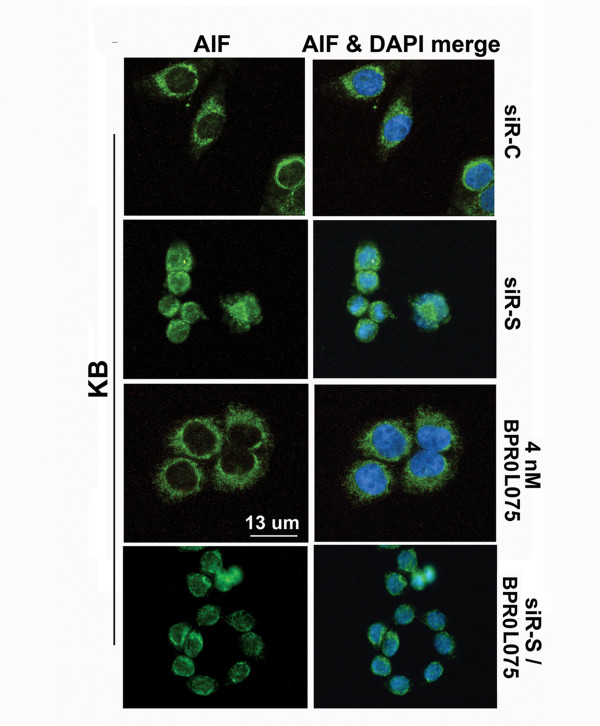
**Down-regulation of survivin with/without BPR0L075 induced the translocation of AIF in KB cells**. KB cells were treated with siR-C (scramble control), siR-S (survivin specific), 4 nM of BPR0L075 or siR-S/BPR0L075 combination for 72 h. AIF was detected using anti-AIF antibody and FITC-conjugated anti-rabbit IgG antibody.

DNA fragmentation can be induced by caspase-independent mechanisms. Apoptosis-inducing factor (AIF) is a protein which induces large-scale DNA fragmentation during caspase-independent apoptosis [31,32]. The possibility that siRNA-mediated down-regulation of survivin would lead to the translocation of AIF was explored. KB cells were treated with siR-C, siR-S, or drug BPR0L075 alone, and AIF expression was examined by immunofluorescence microscopy using an anti-AIF antibody. The cytoplasm of cells treated with siR-C for 48 h was stained green with a ring-like pattern around the nucleus by the anti-AIF antibody, as was the cytoplasm of cells treated with BPR0L075 (Figure 6). In marked contrast, it was the nucleus that was stained green by the anti-AIF antibody in cells treated with the siR-S against survivin, suggesting that AIF translocated from cytoplasm to the nucleus when survivin was targeted with siRNA (Figure 6). Cells treated with a combination of siR-S and BPR0L075 induced similar phenotype to siR-S mono-treatment (Figure 6). Thus, caspase-independent cell death could be induced by down-regulation of survivin in KB cells.

## Discussion

Studies suggest that survivin contributes to chemo-resistance and enhances cell survival through two potential mechanisms: 1) the inhibition of apoptosis by blocking activated caspases [18,22], and 2) by stabilizing the microtubule network to avert cell catastrophe [33]. These two mechanisms exert different weights in the contribution to cancer cell survival, depending on the therapeutic agent used and possibly the cancer type. For example, in lung cancer, Normura et al. demonstrated that inhibition of apoptosis mediated by survivin contributed to cisplatin-resistance [34]. In another study, Zhang et al. showed that that adenovirus-mediated inhibition of survivin expression actually sensitized human prostate cancer cells to paclitaxel [35]. Their studies also suggested that survivin mediates resistance to paclitaxel through suppression of caspase-mediated apoptosis [15]. However, another study revealed that survivin is required for stable checkpoint activation in paclitaxel-treated HeLa cells [23]. Down-regulation of survivin by siRNA abrogated the ability of cells to sustain paclitaxel-induced mitotic arrest [23]. In addition, down-regulation of survivin alone did not induce massive death in HeLa cells [23]. Therefore, question remains as to whether survivin plays a dominant role in processing cytokinesis or inhibiting caspases activity in cells treated with anti-mitotic compounds. Interestingly, survivin-induced resistant to microtubule de-stabilizers such as *Vinca *alkalodis, cochicine and Combretastatin A-4 related compounds in cancers was rarely demonstrated in the past [22]. In our study, we found that survivin counteracts the therapeutic effect of microtubule de-stabilizer, BPR0L075, by stabilizing tubulin polymers.

BPR0L075 is a novel anti-mitotic compound and its mechanisms of action have been published elsewhere [11,12]. In brief, BPR0L075 is a heterocyclic Combretastatin A-4 (CA-4) analog that inhibits tubulin polymerization by binding to tubulin at the colchicine-binding site [11,12]. In the current study, up-regulation of survivin in response to BPR0L075 was observed in both p53-wildtype KB and p53-mutated HONE-1 cancer cells. In addition, the baseline expression of survivin was also increased in KB-derived BPR0L075-resistant KB-*L30*, as compared to its drug-sensitive parental cells. Therefore, one important aim of our study was to investigate whether survivin could be potentially important in sensitivity to the microtubule-destabilizing compound, BPR0L075, especially in BPR0L075-resistant cancer cells. Here, we demonstrate that down-regulation of survivin by siRNA induced hyper-sensitivity to BPR0L075 in KB and partially restored sensitivity to the drug in BPR0L075-resistant KB-*L30 *cells. These results suggested for the first time that survivin plays an important role in the sensitivity and resistant to the microtubule de-stabilizer, BPR0L075. Furthermore, p53 is widely believed to be a negative regulator of survivin expression [25,36-41]. However, our current results indicate that whether p53 plays a definite role in survivin regulation under various chemo-stresses remains agurable. In our study, dose-dependent co-expression of p53 and survivin in response to BPR0L075 was shown in p53-wildtype KB cells. Interestingly, co-incubation of a p53-inhibitor, pifithrin-α, was unable to increase the level of survivin expression in BPR0L075-treated cells. In addition, over-expression of survivin in response to BPR0L075 was also observed in p53-mutated HONE-1 cells. Taken together, our study suggested that BPR0L075 induces overexpression of survivin through a p53-independent pathway.

It has been reported that VEGF-induced expression of survivin preserved the microtubule integrity and stabilization of the microtubule networks subsequently induced drug-resistance to both CDDP and paclitaxel in HUVEC cells [33]. A role for altered microtubule polymer levels in vincristine resistance of acute lymphoblastic leukemia has also been demonstrated *in vivo *[26]. In the present study, increased tubulin polymer levels were observed in the BPR0L075-resistant KB-*L30 *cells as compared to its drug-sensitive parental cells. We also demostrated that down-regulation of survivin de-stabilizes tubulin polymers in both KB and KB-*L30 *cancer cells. In addition, the level of acetylated α-tubulin was synergistically reduced in the survivin/BPR0L075 combination therapy as compared to the monotherapy in KB cells. Thus, the over-expression of survivin in response to the microtubule de-stabilizer BPR0L075 reflects one of the possible cell survival mechanisms by stabilizing microtubule. It is also worth noting that over-expression of survivin was observed in both colchicine- and vincristine-treated KB cells (data not shown). In addition, KB-*L30 *cells are relatively more sensitive to paclitaxel (microtubule stabilizer) and at the same time more resistant to both colchicine and vincristine (microtubule de-stabilizers), as compare to their parental cells (data not shown). Interestingly, Ong et al's study also revealed that a vincristine-resistant xenograft with high levels of polymerized tubulin was relatively sensitive to the microtubule-polymerizing drug paclitaxel [26]. Indeed, further investigations are needed to determine the relationships between the survivin-interfered microtubule dynamics and the resistance to various microtubule de-stabilizers in depth.

Although various studies reveal that survivin interferes with cytokinesis and inhibits caspases during apoptosis, the question remains as to whether survivin plays a dominant role in regulating cell cycle or inhibiting cell death. In fact, down-regulation of survivin did not induce massive cell death in HeLa cells [23]. However, the process of cytokinesis was heavily impaired by the same treatment [23]. In the present study, down-regulation of survivin did not induce cell death in both KB and KB-*L30 *cells under BPR0L075-free conditions. In accord with the results from cell viability study, down-regulation of survivin did not induce caspase-3/-7 activities in both KB and KB-*L30 *cells cultured under drug-free conditions. It is widely believed that anti-mitotic compounds induce mitotic arrest by activating the spindle checkpoint and these molecular changes subsequently activate caspase-dependent apoptosis. Our previous study agreed with that hypothesis as the microtubule de-stabilizer, BPR0L075, induced activation of caspase-3 in various cancer cell lines [12]. Interestingly, down-regulation of survivin did not enhance caspase-3/-7 activities in the survivin/BPR0L075 co-treated KB cells, given that the microtubule dynamics were heavily impaired in the same treatment. These results suggested that survivin functions predominantly in microtubule stabilization or mitotic progression, as opposed to inhibition of caspase-dependent cell death in KB cells under BPR0L075-free conditions. However, down-regulation of survivin induced caspase-3/-7 activities in the survivin/BPR0L075 co-treated KB-*L30 *cells. Therefore, whether survivin interferes with the caspase-3/-7 during BPR0L075 treatment seems to be cell type dependent. Furthermore, incomplete restoration to the drug sensitivity in survivin down-regulated KB-*L30 *cells indicates that other important drug resistance mechanisms may co-exist.

As we mentioned earlier, it is believed that anti-mitotic compounds induce mitotic arrest by activating the spindle checkpoint and these molecular changes subsequently activate caspase-dependent apoptosis. However, our current study reveals that down-regulation of survivin synergistically affects the microtubule dynamics and cell viability without significant induction in caspases activity. In addition, increased DNA fragmentation and nucleus degradation were observed in the survivin/BPR0L075 combination treatments, as compared to the BPR0L075 monotherapy. Therefore, down-regulation of survivin together with BPR0L075 may initiate caspase-independent DNA fragmentation and cell death in KB cells. During one form of caspase-independent apoptosis, apoptosis-inducing factor (AIF) translocates from the mitochondria to the nucleus [42-44]. AIF is a flavoprotein that is normally confined to the mitochondrial intermembrane space, but induces chromatin condensation and fragmentation of DNA into high molecular weight forms of >50 kb when it translocates to the nucleus [31,32]. It has been suggested that survivin interferes with the translocation of AIF. In fact, translocation of AIF from the mitochondria to the nucleus was shown in YUSAC2 melanoma cells treated with a cell-permeable dominant-negative survivin protein [45]. Furthermore, involvement of AIF in nocodazole (microtubule de-stabilizer)-induced caspase-independent mitotic cell death was also demostrated perviosuly [46]. In Niikura et al.'s study, treatment with nocodazole or paclitaxel induced kinetochore-microtubule detachement, mitotic arrest and subsequent AIF-involved caspase-independent cell death in cancer cells [46]. In our study, translocation of AIF from the cytoplasm to the nucleus following the down-regulation of survivin indicates that survivin inhibits caspase-independent apoptosis in KB cells. Thus, both caspase-dependent and caspase-independent DNA fragmentation could be co-operated in KB cells during survivin-targeted BPR0L075 combination treatments. These results suggested that down-regulation of survivn together with microtubule de-stabilizers may induces caspase-independent mitotic cell death, possible through AIF activation. However, our study could not determine the rate of AIF activity between survivin monotherapy and survivin/BPR0L075 combination. Further investigation is needed to determine the level of AIF activities between different treatments.

## Conclusion

Drug resistance is a common problem in the management of neoplastic diseases. The effectiveness of a single anti-cancer agent is limited by the fact that drug resistance mechanisms can be introduced by cancer cells during cell evolution or re-arrangement of tumor micro-environment. In clinical situations, rather than treating patients with a single anti-cancer agent, combination therapies are preferred. BPR0L075 is a tubulin-targeting compound capable of inducing cytotoxic effect among various MDR-positive/-negative cancers both *in vitro *and *in vivo*. Down-regulation of survivin with an anti-sense survivin gene enhances the sensitivity to the DNA damaging agent, cisplatin, which in KB have been demonstrated both *in vitro *and *in vivo *previously [14]. However, the underlying molecular mechanisms of the drug resistant to anti-mitotic agents, especially for the microtubule de-stabilizing compounds, have not been discussed in detail. Here, we have shown that survivin counteracts the therapeutic effect of microtubule de-stabilizer, BPR0L075, by stabilizing tubulin polymers in human KB oral carcinoma cells. Since survivin plays an important role in the sensitivity of microtubule de-stabilizing agents, the use of survivin-targeted agents such as oxaliplatin and SPC3042 (Santaris Pharma) in anti-mitotic combination therapy maybe of clinical benefit [47,48]. Also, unlike other potential therapeutic targets, survivin is expressed in few adult tissues. Thus, survivin-specific combination therapy is likely to produce few adverse effects.

## Methods

### Drug

The compound BPR0L075 was synthesized at the Division of Biotechnology and Pharmaceutical Research, National Health Research Institutes, Zhunan, Taiwan, ROC. BPR0L075 was obtained in 72% yield from 6-methoxyindole and 3,4,5-trimethoxybenzoyl chloride. The detailed synthetic method was previously published[11]

### Cell lines, antibodies and reagents

The human oral carcinoma cells (KB) were purchased from the American Type Culture Collection (ATCC, Manassas, VA). KB and human nasopharyngeal carcinoma (HONE-1) cells were cultured in RPMI 1640 medium (Gibco, Grand Island, NY), supplemented with 5% fetal bovine serum, penicillin (100 U/mL), streptomycin (100 μg/mL) and L-glutamine (0.29 mg/mL), at 37°C. The antibodies used in this study included: a mouse anti-α Tubulin antibody (Upstate Cell signaling, Lake Placid, NY), a mouse anti-β Tubulin antibody (BD PharMingen, Franklin Lakes, NJ), a mouse anti-Actin antibody (Santa Cruz Biotechnology, Santa Cruz, CA), a rabbit anti-Survivin antibody (R&D Systems, Minneapolis, MN) and a rabbit anti-AIF antibody (R&D Systems, Minneapolis, MN), a mouse anti-Bcl2 antibody (Santa Cruz Biotechnology, Santa Cruz, CA).

### Establishment of the KB-derived BPR0L075-resistant cell line

Human oral carcinoma cells (KB) were cultured in RPMI 1640 medium as previously described. BPR0L075-resistant cells were established from KB cells by exposure to increasing concentrations of BPR0L075. Briefly, KB cells were initially incubated in completed medium containing 5nM of BPR0L075 that yielded 40% cell survival for a period of 3~4 weeks, and the cells that proliferated were repeatedly subcultured in completed medium containing increasing concentrations of the drug (a 20% increment each time). Cells that grew exponentially in the presence of 30 nM of BPR0L075 were obtained and subcloned by dilution plating in 48-well plates. Individual clones were isolated. For maintenance, these subclones were cultured under conditions similar to those used for KB, except for an addition of BPR0L075 (30 nM).

### RT-PCR

Total RNA was extracted with using TRIzol reagent (Invitrogen, Carlsbad, CA) and complementary DNA was synthesized from RNA with the SuperScript™ First-Strand Synthesis System (Invitrogen, Carlsbad, CA). Polymerase chain reaction was performed with target-specific primers. Survivin forward primer: 5' ATGGGTGCCCCGACGTT; Survivin reverse primer: 5' TCAATCCATGGCAGCCAG; GAPDH: 5' ACCACAGTCCATGCCATCAC and GAPDH reverse primer: 5' TCCACCACCCTGTTGCTGTA.

### Real-time reverse transcription-polymerase chain reaction (Real-time PCR)

Expression level of Survinin transcript was determined by real-time reverse transcriptase (RT)-polymerase chain reaction (PCR) using a LightCycler instrument (Roche, Indianapolis, IN). Primers and Taqman probes were designed by Probe Finder™ . Taqman probes were from the Universal Probe Library: Survinin [49] and hGAPDH [17]. Specific primers with following sequences were used: Survinin forward, 5' GCCCAGTGTTTCTTCTGCTT; Survivin reverse, 5'CCGGACGAATGCTTTTTATG; hGAPDH forward, 5' AGCCACATCGCTCAGACAC and hGAPDH reverse, 5' GCCCAATACGACCAAATCC. The real-time PCR condition was as follows: 1 cycle of initial denaturation at 95°C for 10 min, 45 cycles of amplification at 95°C for 10 s, 60°C for 30 s, and 72°C for 1 s, with a single fluorescence acquisition. GAPDH gene was used as an internal control.

### siRNA

Target-validated siRNA oligos (Santa Cruz Biotechnology, Santa Cruz, CA) were transfected into cells using the Lipofectamine-2000 reagent (Invitrogen, Carlsbad, CA). Briefly, cells were seeded onto 96-well plates or chamber-slides, and cultured overnight in 100 μl of antibiotic-free RPMI media. siRNA oligomers (8 pmol in 0.4 μl) were diluted in 25 μl of Opti-MEM^® ^I medium (Invitrogen, Carlsbad, CA) without serum, and then mixed with 0.2 μl of Lipofectamine-2000 transfection reagent for 25 min at room temperature. Cells were overlaid with the transfection mixture, and incubated for various times.

### SDS-PAGE and Western Blot Analysis

Cells were lysed with ice-cold lysis buffer (10 mM Tris, 1 mM EDTA, 1 mM DTT, 60 mM KCl, 0.5% NP-40 and protease inhibitors). Total cell lysates, fractions of supernatant or pellet were resolved on 10% and 12% polyacrylamide SDS gels under reducing conditions. The resolved-proteins were electrophoretically transferred to PVDF membranes (Amersham Life Science, Amersham, U.K.) for Western blot analysis. The membranes were blocked with 5% non-fat milk powder at room temperature for two hours, washed twice with PBST (1% Tween) and then incubated with primary antibody for 90 minutes at room temperature. The membranes were washed twice with PBST then subsequently incubated with a horseradish peroxidase-conjugated secondary antibody (dilution at 1:10000, Santa Cruz Biotechnology, Santa Cruz, CA). Immunoreactivity was detected by Enhanced Chemiluminescence (Amersham International, Buckingham, U.K.) and autoradiography.

### MTT cell viability assay

3 × 10^3 ^cells in 100 μL of drug-free culturing medium were seeded onto 96-well plates for 24 hours before treatments. Cells were then treated with various concentration of BPR0L075 for 72 hours. 25 μL of MTT (5 mg/mL) was added into each sample and incubated for 4 hours, under 5% CO_2 _and 37°C. 100 μL of lysis buffer (20% SDS, 50% DMF) was subsequently added into each sample and further reacted for 16 hours. IC_50 _value resulting from 50% inhibition of cell growth was calculated graphically as a comparison with control growth.

### Immunofluorescent microscopy

Cells were cultured in 8-well chamber slides, fixed with 4% paraformaldehyde, and permeabilized with TPBS. They were incubated with primary antibody for 60 min at room temperature followed by FITC-conjugated secondary antibody. Slides were examined by microscopy using an Olympus BX50 microscope (Olympus optical co., LTD, Tokyo, Japan). Images were taken on the Olympus microscope with the use of software CoolSNAP (Roper Scientific, Inc.)

### Real-time Caspase-3/-7 activity imaging and caspase-3/-7 activity assay

Caspase-3/-7 activity was analyzed with a MagicRed™-DEVD Caspase Detection Kit (Immunochemistry Technologies LLC, Bloomington, MN). Briefly, cells were cultured in chamber-slides and incubated with test agents. Cells were then incubated with caspase substrate MR-(DEVD_2_) in culture medium for 60 min, and then with Hoechst 33342 stain for 15 min. Cells were viewed with a UV-enabled inverted-microscope at an excitation wavelength of 540 nm – 560 nm and emission at 610 nm.

Quantitative analysis of caspase-3/-7 activity was performed on 96-well plates. Cells were cultured on 96-well plates and incubated with test agents. Treated-cells were then incubated with caspase substrate MR-(DEVD_2_) in culture medium for 90 min. Caspase-3/-7 activity was measured with a 96-well plate reader at an excitation wavelength of 590 nm and emission at 615 nm.

### Visualization of apoptosis by the TUNEL assay

Cells were seeded and cultured in 8-well chamber-slides, and treated with various treatments. The cells were washed with PBS, fixed with 4% paraformaldehyde for 30 min on ice, and permeabilized with TPBS at room temperature. Apoptotic cells were stained by the TUNEL agent using an In-Situ Apoptosis Detection TMR Kit (Roche Diagnostic, Mannheim, Germany). Cells were counter-stained with DAPI to detect nucleus, and examined by fluorescence microscopy.

## Abbreviations

AIF: apoptosis inducing factor; BPR0L075 and L075: [6-methoxy-3-(3',4',5'-trimethoxy-benzoyl)-1H-indole]; CA-4: combretastatin A-4; IAP: inhibitors of apoptosis; MRP: multidrug resistance protein; siR-C: scramble siRNA oligos; siR-S: survivin-targeted siRNA oligos.

## Competing interests

The authors declare that they have no competing interests.

## Authors' contributions

CHAC performed the in *vitro *studies and drafted the manuscript. HHC performed the quantitative RT-PCR analysis. CCK helped to determine the IC_50 _values of various anti-mitotic compounds in drug sensitive and resistant cells. CYC helped to establish the drug resistant cancer cell line. MSC and HPH participated in synthesizing the anti-mitotic compound, BPR0L075. JYC participated in coordination of the study. All authors read and approved the final manuscript.

## Authors' information

C. H. A. Cheung, *Ph.D. (Post-doctoral research fellow, molecular biologist)*

H. H. Chen, *Ph.D. (Post-doctoral research fellow, molecular biologist)*

C. C. Kuo, *Ph.D. (Assistant-principle investigator, molecular biologist)*

C. Y. Chang, *M.Sc. (Research assistance)*

M. S. Coumar, *Ph.D. (Post-doctoral research fellow, biochemist)*

H. P. Hsieh, *Ph.D. (Principle investigator, biochemist)*

J. Y. Chang, *M.D. (Distinguished investigator, medical oncologist)*

## Supplementary Material

Additional file 1 **Analysis of the cell cycle distribution**. (A) KB cells were treated with various concentrations of BPR0L075 for 24 h. Cells were stained with propidium iodide and subsequent analyzed by flow cytometry. (B) KB and KB-*L30 *cells cultured under BPR0L075-free conditions were stained with propidium iodide and subsequent analyzed by flow cytometry. Click here for file
