# Supplementary figures and images for: Survivin counteracts the therapeutic effect of microtubule de-stabilizers by stabilizing tubulin polymers
Source: Mol Cancer. 2009 Jul 3;8:43. doi: 10.1186/1476-4598-8-43 (PMC2714493; doi:10.1186/1476-4598-8-43)

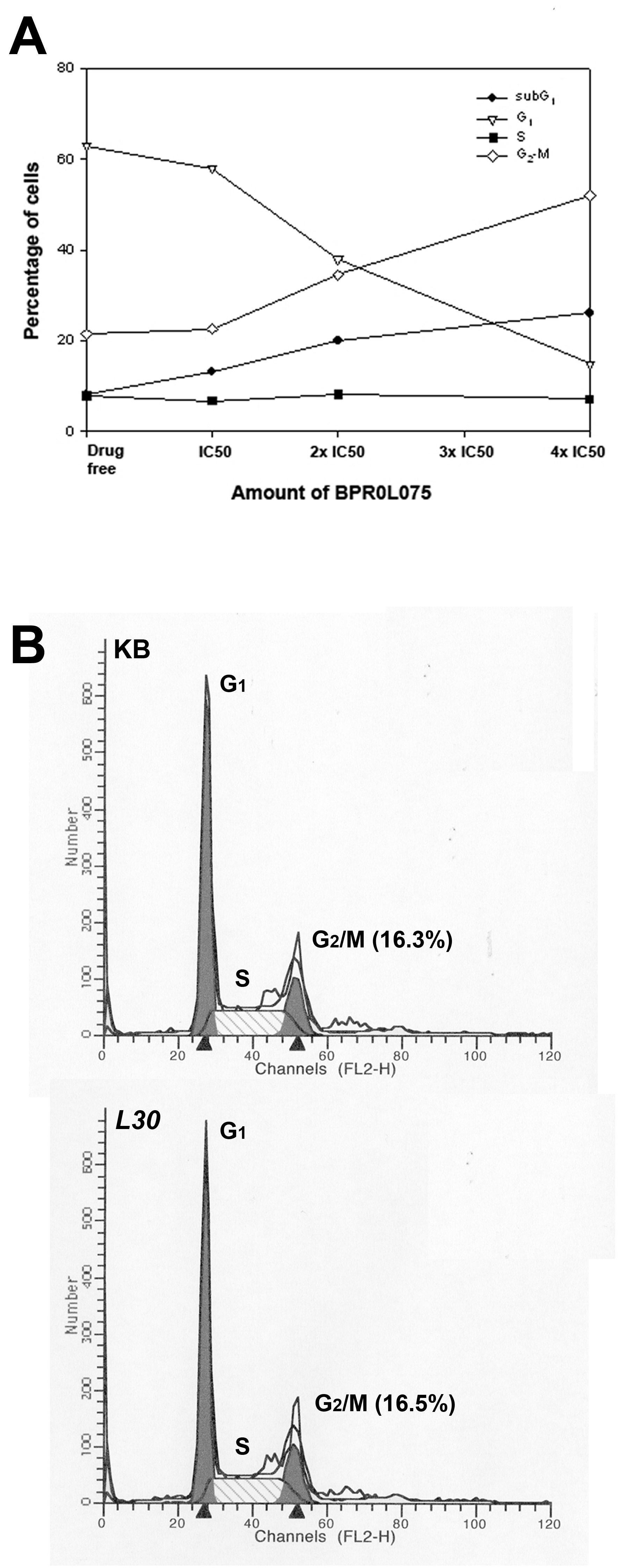

Supplement: Additional file 1 — Analysis of the cell cycle distribution. (A) KB cells were treated with various concentrations of BPR0L075 for 24 h. Cells were stained with propidium iodide and subsequent analyzed by flow cytometry. (B) KB and KB-L30 cells cultured under BPR0L075-free conditions were stained with propidium iodide and subsequent analyzed by flow cytometry. [file 1476-4598-8-43-S1.jpeg]
